# Expression of RAD51 and Its Clinical Impact in Oral Squamous Cell Carcinoma

**DOI:** 10.1155/2020/1827676

**Published:** 2020-03-03

**Authors:** Yuyang Li, Jia Li, Jingchun Sun, Yingkun Liu, Dingkun Liu, Liuyi Du, Bizhou Wang, Weiwei Liu

**Affiliations:** ^1^Department of Dental Implantology, Hospital of Stomatology, Jilin University, Changchun 130021, China; ^2^Jilin Provincial Key Laboratory of Tooth Development and Bone Remodeling, Changchun 130021, China; ^3^Department of Oral and Maxillofacial Surgery, Hospital of Stomatology, Jilin University, Changchun 130021, China; ^4^Department of Prosthodontics, Hospital of Stomatology, Jilin University, Changchun 130021, China

## Abstract

**Purpose:**

To examine the expression of RAD51 in oral squamous cell carcinoma (OSCC) and analyze its connection with pathological grade, clinical stage, and lymphatic metastasis potential.

**Methods:**

For this study, 74 OSCC samples, 15 normal mucosa tissues, and 11 normal skin tissue samples were collected. RAD51 expression was investigated using immunohistochemistry. A follow-up visit was used to assess the prognosis of each patient. We compared RAD51 expression in oral mucosa epithelial cells (OMECs), keratinocytes, and tongue squamous cell carcinoma cells (TSCCs) by Western blot analysis.

**Results:**

RAD51 expression was higher in tumor cells than in normal mucosal tissues. In addition, RAD51 expression was associated with higher tumor differentiation (*P* < 0.05). Also, RAD51 expression was higher (*P* < 0.05). Also, RAD51 expression was higher (*P* < 0.05). Also, RAD51 expression was higher (

**Conclusion:**

A strong positive correlation was found between RAD51 expression and the degree of malignancy in OSCC patients, suggesting that RAD51 could be an excellent prognostic indicator for OSCC patients.

## 1. Introduction

Oral squamous cell carcinoma (OSCC) accounts to more than 90% of all oral malignancies diagnosed each year. Globally, it is the most common form of cancer that arises from the mucosal membranes of the oropharynx and mouth with more than 275,000 new cases diagnosed each year and 125,000 deaths attributed to this malignancy each year worldwide [1]. Due to the rich lymphatic network in the maxillofacial region, OSCC displays a high metastatic potential with more than 40% of OSCC patients developing cervical lymph node metastases within two years of their initial diagnosis [2]. Once the malignancy spreads to the surrounding lymph nodes, patient survival rates decline significantly with only 40-50% of individuals surviving five-years after the initial diagnosis. However, the survival rate for patients without metastases is 90%, suggesting that the key to improving survival rates is the early diagnosis and prompt treatment of the disease. With this in mind, scientists have identified several biomarkers that could be useful for identifying early-stage disease in symptomatic patients or high-risk individuals [3].

RAD51 is a 339-amino acid that plays an essential role in repairing DNA double-strand breaks. When the double-stranded structure of DNA is injured, RAD51 exploits a sister DNA molecule as a template that allows for homologous recombination (HR) in the damaged region, which maintains the stability of the genes [4]. While RAD51 displays a protective role, scholars have found that RAD51 is overexpressed in several tumor types, including breast, pancreatic, head and neck, prostate, non-small cell lung, and esophageal cancers [5, 6]. Recently, Yuan et al. [7] discovered that high RAD51 expression was directly associated with increased chemo- and radio-resistance, along with dismal outcomes and prognoses in breast cancer patients. In another study, Chen et al. [8] revealed that RAD51, which was overexpressed in patients with cervical cancer, promoted the differentiation of cancer cells from the G0/G1 to S phase. Interestingly, inhibition of RAD51 increased the sensitivity of the cancer cells to radiotherapy, which is a potential therapeutic strategy that requires further exploration. Furthermore, the results from a recent meta-analysis revealed that high RAD51 expression could increase the risk of patients developing head and neck tumors [9].

The current literature shows that radiotherapy after surgery is an ideal treatment for squamous cell carcinoma of the head and neck for improving the overall survival and quality of life for patients. However, radiotherapy causes off-site injury to healthy tissues, which can lead to unwarranted inflammation or the development of mucosal ulcers [10]. The radiation will also negatively impact the epidermal cells and oral mucosa epithelial cells, yet the adverse effects associated with these cells are less severe than the mucosa [11]. Therefore, we believe that RAD51 may play an important role in resisting radiation-induced damage.

Currently, limited studies have assessed the role of RAD51 in OSCC. In this study, we aimed to evaluate the potential use of RAD51 as a prognostic indicator of OSCC. To do this, we examined RAD51 expression in OSCC, oral mucosa, and skin tissue samples and analyzed its association with lymphatic metastasis. We also compared RAD51 expression in oral mucosa epithelial cells (OMECs), keratinocytes, and tongue squamous cell carcinoma cells (TSCCs) to provide a theoretical basis for the clinical use of RAD51 as a prognostic biomarker for oral cancer.

## 2. Material and Methods

### 2.1. Patient Samples

The study was approved by the Ethics Committee of Jilin University. Prior to the study, all patients provided written informed consent. For this study, the Department of Oral Pathology provided 74 OSCC tissue samples, 15 adjacent normal tissue samples, and 11 normal skin tissue samples from patients treated at the Hospital of Stomatology, Jilin University (Changchun, China), between 2013 and 2017. Those patients with a history of diabetes, hypertension, or systemic, metabolic, and immunological diseases were excluded from the study. In addition, the consumption of alcohol and cigarette smoking were also exclusion criteria. None of the patients received prior treatments, including surgery, radiotherapy, or chemotherapy before the surgery at the Hospital of Stomatology, Jilin University. Of the patients, 55 were male (74.3%) and 19 were female (25.7%) with a mean age of 57.2 years (range: 33-81). Histologically, 21 cases were well-differentiated, 32 were moderately differentiated, and 21 were poorly differentiated cases of OSCC. A total of 28 cases (14 + 14, 37.8%) had early-stage disease (I + II), while 46 cases (22 + 24, 62.2%) had advanced-stage disease (III + IV). Furthermore, 21 cases (28.4%) had lymphatic metastases whereas 53 cases (71.6%) had no lymphatic metastases (Table 1). Patient samples were collected after surgery and sectioned at 4 *μ*M thickness after being fixed in 10% formalin, dehydrated, and paraffin embedded. The sections were adhered to slides and used for histological studies.

### 2.2. Immunohistochemistry

The OSCC and normal tissue samples were subjected to immunohistochemistry using a commercial kit (MXB Biotechnologies, Fuzhou, China). The optimal concentration of polyclonal antibodies against RAD51 (ABclonal Biotech Co., Ltd, Wuhan, China) was 1 : 100. According to the protocol outlined by the manufacturer, the sections were deparaffined in xylene, graded alcohol, and tap water. Next, the sections underwent microwave antigen retrieval in a citric acid solution (pH 6.0) for 10 min before incubating in the endogenous peroxidase blocker (3% H_2_O_2_) for 10 min. After washing with PBS, the sections were incubated with normal sheep serum at room temperature for 20 min. Then, the primary antibody diluted in PBS was added for an overnight incubation at 4°C. The sections were washed in PBS and incubated with sheep anti-rabbit IgG for 10 min. After washing in PBS, the sections were incubated in biotin-labeled streptavidin (10 min) and subjected to a color reaction using DAB kit (MXB Biotechnologies, Fuzhou, China) for 3 min and counterstaining in hematoxylin for 1 min. Next, the sections were processed by hydrochloric acid alcohol, and ammonia. Finally, the sections were dehydrated in graded ethanol solutions, cleared in xylene, and mounted with neutral balsam using coverslips.

### 2.3. Immunohistochemical Scoring

The stained sections were viewed and scored under a light microscope by two independent investigators. Each section was evaluated at five fields at 400x magnification. The presence of a clearly visible yellow or brown precipitation was considered an immunoreaction, and each section was scored according to the degree of positive staining and the staining intensity. The samples were classified as negative (0 as 0-10% positivity) or positive (1 as 10-25% positivity, 2 as 25-50% positivity, 3 as 50-75% positivity, and 4 as 75-100% positivity). Expression intensity was evaluated semiquantitatively without prior knowledge of any clinical information using a four-level system (0 as negative, 1 as weak, 2 as moderate, and 3 as strong) [12]. The staining index for each section was then reached by multiplying the positive cell score by the intensity score to obtain a final score, which was an average of five scores. For statistical analysis, the samples were categorized into two groups: negative (score ≤ 5) and positive (score > 5) [13].

### 2.4. Cell Culture

The TSCC cell lines, CAL-27 and SCC-9, were purchased from ATCC (Manassas, VA, USA). Human keratinocytes (HaCaT) were provided by Professor Hongchen Sun (Department of Oral Pathology, Hospital of Stomatology, Jilin University). OMECs were isolated from the excessive mucosal tissues of a healthy donor (male, 23 years) in an orthognathic surgery from the Department of Oral and Maxillofacial Surgery, Hospital of Stomatology, Jilin University. The donor provided written informed consent prior to this study. The mucosal tissues were washed with PBS, and the epithelial part was isolated and cut into small fragments (1 mm^3^) before digesting in 2.5 g/L Dispase II (Solarbio Science & Technology Co., Ltd., Beijing, China). After washing in PBS, the epithelium was digested in the mixed liquor containing 0.25% trypsin (Invitrogen) and 0.03% EDTA (Invitrogen) and blocked in DMEM to prepare monoplast suspension.

CAL-27 cells were maintained in RPMI-1640 (Invitrogen) and supplied with 10% fetal bovine serum (FBS; PAA Laboratories, Pasching, Austria), 100 U/mL penicillin, and 100 mg/L streptomycin. SCC-9 cells were incubated in DMEM/F-12 (Invitrogen) with the supplements of 10% FBS and 400 *μ*g/L hydrocortisone (Sigma). HaCaT and oral mucosa epithelial cells were cultured in Keratinocyte Serum Free Medium (K-SFM; Gibco). All of the cells were maintained at 37°C in a humidified incubator with 5% CO_2_. The experiments were carried out using cell growth in the logarithmic phase.

### 2.5. Western Blot Analysis

Cells were washed with PBS and lysed in RIPA buffer (Millipore, Billerica, MA). Protein concentration was determined with the bicinchoninic acid protein assay. First, 40 *μ*l of total protein was resolved using a 10% SDS-PAGE and transferred onto PVDF membranes (Bio-Rad, Hercules, CA, USA). After blocking with 5% skim milk powder for 1.5 h, the membranes were incubated with primary antibodies for RAD51 (Cell Signaling Technology, Shanghai, China) at 4°C for overnight. After washing, the membranes were treated with the appropriate horseradish peroxidase-conjugated secondary antibody and visualized using the enhanced chemiluminescence systems (GE Healthcare, London, UK). *β*-Actin was used for normalization.

### 2.6. Clinical Follow-Up Visits

Follow-up visits were carried out with select patients for more than five years after surgery. During the follow-up visits, patient characteristics, treatment plans, and cases of relapse were recorded.

### 2.7. Statistical Analysis

All statistical analyses were performed using SPSS version 18.0 (IBM, Chicago, IL, USA). Comparisons between groups were assessed using the one-way analysis of variance (ANOVA), Student's *t* test, or *χ*^2^ test. *P* values of less than 0.05 were considered statistically significant.

## 3. Results

### 3.1. RAD51 Expression between the OSCC and Healthy Tissue Samples

**Figure 1:**
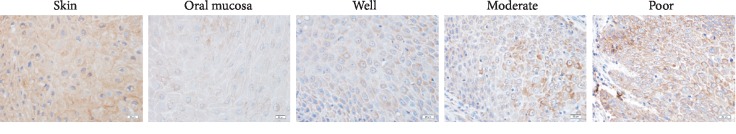
Expression of RAD51 in OSCC tissues (×400). RAD51 expression was lower in normal tissues than in OSCC tissues (*P* < 0.05). RAD51 was highest in the poorly differentiated OSCC tissues and lowest in the well-differentiated OSCC when compared with the healthy control tissues (*P* < 0.05).

RAD51 expression was mainly localized on the cytoplasm and a few on the nucleus of cells. The between-group comparisons are shown in Table 2. RAD51 expression was significantly stronger in OSSC tissues when compared with normal tissues, and RAD51 expression levels were positively correlated with the disease stage (*P* < 0.05; Figure 1), suggesting that RAD51 expression could be potentially used to estimate the prognoses of OSCC patients. In addition, RAD51 expression was also higher in OSCC patients with lymphatic metastases (*P* = 0.027). Lastly, according to the TNM stage, we found that RAD51 expression was higher in patients with advanced-stage disease (III or IV) when compared with those patients with early-stage (I or II) OSCC (*P* = 0.055), yet this finding was not statistically significant.

### 3.2. RAD51 Expression Levels in TSCC Cells Were Higher than Those in Oral Mucosa Epithelial Cells and Lower than those in HaCaT Cells

**Figure 2:**
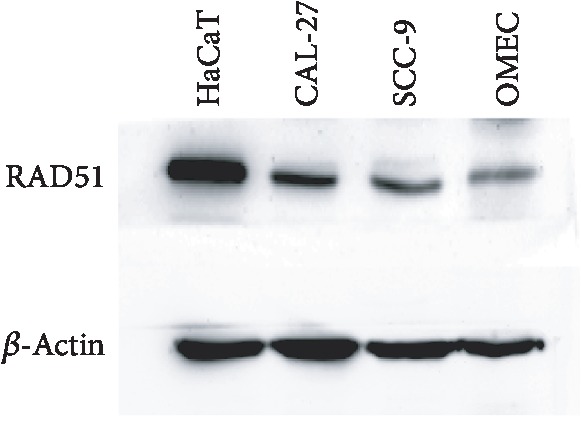
Expressions of RAD51 in different cell lines. Western blot analysis showed the RAD51 expression was highest in the HaCaT cells, followed by TSCC cells (CAL-27 and SCC-9), and oral mucosal epithelial cells.

Western blot analysis was used to assess the RAD51 expression levels among the TSCC cells, OMECs, and skin cells. RAD51 was differentially expressed in all of the cell lines. As shown in Figure 2, RAD51 expression levels were highest in the in the skin keratinocytes (HaCaT), followed by the TSCC cells (CAL-27 and SCC-9). The OMECs showed the lowest levels of RAD51 expression. The reparability of TSCC cells was stronger compared to OMECs.

### 3.3. Follow-Up Visits

There were total 39 patients who met the time standards, of which one patient lost touch and 38 patients provided the relevant information (Table 3). Of the 38 patients, eighteen were deceased by the follow-up and 19 patients had relapsed. RAD51 expression was significantly higher in the samples from patients who were deceased by the follow-up visit (*P* = 0.036). Also, there was a high tendency of RAD51 expression in patients who have recurrent disease when compared with patients with nonrecurrence (*P* = 0.052), yet this finding was not statistically significant. In order to further explore the function of radiotherapy in postoperative patients, we analyzed the cases of disease recurrence. Results showed that the relapse rate in the radiotherapy group (43.3%) was lower than that in patients who did not receive radiotherapy (75%, *P* = 0.047). In recurrent cases, we also compared RAD51 expression among the well, moderately, and poorly differentiated groups. In all the groups, including the well (*P* < 0.01), moderately (*P* = 0.073), and poorly differentiated (*P* = 0.024), RAD51 expression was higher in those patients with the relapsed disease when compared with those patients without relapsed disease. Overall, high expression of RAD51 indicated a poor prognosis and a high possibility of disease recurrence in OSCC patients.

## 4. Discussion

In recent years, RAD51 expression has been detected in several types of cancer, including mainly breast cancer [14, 15], cervical cancer [15, 16], and ovarian cancer [17]. The homozygous gene RAD51 GG was found to be most often in breast cancer patients [16], while RAD51 G172T has the most clinical significance in cervical cancer [18, 19]. In this study, we investigated the possible link between RAD51 expression and OSCC and found that RAD51 was expressed higher in OSCC patients. In addition, we discovered that RAD51 expression was significantly higher in poorly differentiated tissues compared with moderately or well-differentiated tissues (Figure 1, Table 3) and that patients with advanced-stage disease showed stronger levels of RAD51 when compared with patients with early-stage OSCC (*P* = 0.055). These findings suggest that RAD51 may be a viable biomarker for staging OSCC in the future [20].

Overexpression of RAD51 can result in the formation of genotoxic RAD51 protein complexes on undamaged chromatin, which will decrease the efficiency of homologous recombination [21]. Also, RAD51 overexpression can lead to the enhancement of cell growth inhibition and apoptotic induction, resulting in tumor progression [22]. Furthermore, RAD51 can protect tumor cells from radiation-induced damage. Previously, An et al. [23] used T0070907 (T007), one kind of peroxisome proliferator-activated receptor gamma (PPAR*γ*), to weaken the expression of RAD51 in cervical cancer and disturb the mitosis of tumor cells. They found that the agent also increased the sensitivity to radiation therapy. In another study, Liu et al. [24] knocked-down the RAD51 gene in triple-negative breast cancer (TNBC) cells, which were found to inhibit cell proliferation. In addition, the cancer stem cells (CSCs) in the TNBC could resist poly ADP-ribose polymerase (PARP) inhibitors. However, when the RAD51 gene was knocked-down in the CSC, its sensitivity to PARP inhibitors and radiation therapy increased, which effectively inhibited tumor growth [25].

In the current study, we analyzed a potential connection between RAD51 expression and OSCC. Our findings demonstrated that high expression of RAD51 was indicative of poor patient prognoses (Table 3). In immunohistochemistry studies, RAD51 expression in the well-differentiated group was lower than the moderately and poorly differentiated groups. Compared to those patients who did not have a recurrence of OSCC by the time of the follow-up visit, whatever in the well (*P* < 0.01), moderately (*P* = 0.073), or poorly (*P* = 0.024) differentiated group, higher levels of RAD51 could be shown in the recurrent case samples. All of the above findings indicated that increased expression of RAD51 was closely related to the occurrence, development, and relapse of OSCC.

Furthermore, the upregulation of DNA repair protein, like RAD51, could reduce the radiation-induced DNA damage [26]. Considering the derivation of OSCC, we compared the expression levels of RAD51 among TSCCs, OMECs, and keratinocytes and found that RAD51expression was highest in the skin, followed by the OSCC, and oral mucosa (Figure 1, Table 2). Cell-based experiment showed similar findings (Figure 2), indicating that skin tissue has a stronger ability to resist radiation-induced damage. This explains why oral mucosa is ulcerogenic during the radiotherapy [10], while the effects on the skin are less pronounced. Nonetheless, skin tissues need more time to recover from the radiation-induced damage than oral mucosal tissues [27], yet the exact mechanisms remain to be elucidated.

In addition, high RAD51 expression was found to be correlated with increased metastatic potential. Recently, Mahdi et al. [28] examined RAD51 expressions in primary ovarian tumors and metastatic and discovered significantly higher expression in the metastatic tumors. High expression of RAD51 in breast cancer was previously shown to increase the risk of brain metastases and micrometastases. However, the knockdown of RAD51 decreased the metastatic potential of breast cancer [29]. Scholars have confirmed that high RAD51 expression could function as a critical factor in the promotion of lymphatic metastases from tumors [30, 31]. As a confirmation of that finding, this study showed that RAD51 expression was significantly higher in OSCC patients with lymphatic metastases (*P* < 0.05).

This study is the first to report on the high expression of RAD51 in OSCC patients, which was compared with the oral mucosa and skin. While the RAD51 protein has a recovery effect, it can also enhance the antidamage and invasion abilities of tumor cells because of its nonselective properties. Therefore, RAD51 may be viewed as an important protein for future targeted therapies. Overall, we found that RAD51 was an excellent prognostic indicator for patients with OSCC.

## Figures and Tables

**Table 1:** Gender and clinical data about OSCC patients.

Variables	# of patients	% of patients
Gender		
Male	55	74.3
Female	19	25.7
Histological grade		
Well	21	28.4
Moderate	32	43.2
Poor	21	28.4
Clinical stage		
I	14	18.9
II	14	18.9
III	22	29.7
IV	24	32.5
Lymphatic metastasis		
No	53	71.6
Yes	21	28.4

**Table 2:** RAD51 expression in OSCC patients.

Variables	Positive number (%)	*P* value
Gender		0.275
Male	26 (47.3)	
Female	7 (36.8)	
Property		< 0.05
Oral mucosa	3 (20)	
OSCC	33 (44.6)	
Skin	7 (63.6)	
Histological grade		< 0.05
Well	5 (23.8)	
Moderate	14 (43.8)	
Poor	14 (66.7)	
Clinical stage		0.055
I + II	9 (32.1)	
III + IV	24 (52.2)	
Lymphatic metastasis		0.027
Yes	13 (59.1)	
No	20 (38.5)	

**Table 3:** RAD51 expression is linked to dismal patient survival and patient relapse.

Variables	Number of patients (%)	*P* value
Death	18 (47.4)	0.036
Relapse	19 (50)	0.052
Relapse with radiotherapy	13 (43.3)	0.047
Relapse without radiotherapy	6 (75)	
Well-differentiated	3 (15.8)	<0.01
Moderately differentiated	12 (63.1)	0.073
Poorly differentiated	4 (21.1)	0.024

## Data Availability

The data and materials are available on reasonable request.
